# Membrane Complexity and Phase Behavior Dictate the Stability of Membrane‐Inserted A*β*_42_ Hexamers

**DOI:** 10.1002/cphc.70538

**Published:** 2026-08-21

**Authors:** Bastian Ferdinand Bundschuh, Franziska Kley, Yu Wang, Birgit Strodel

**Affiliations:** ^1^ Institute of Biological Information Processing: Structural Biochemistry Forschungszentrum Jülich Jülich Germany; ^2^ Faculty of Mathematics and Natural Sciences Heinrich Heine University Düsseldorf Düsseldorf Germany

**Keywords:** *β*‐barrel, alzheimer’s disease, amyloid‐*β*, molecular dynamics, neuronal membrane

## Abstract

Protein–membrane interactions are vital to Alzheimer’s pathogenesis, as lipid environments modulate both the aggregation and toxicity of amyloid‐*β* (A*β*) peptides. Using atomistic molecular dynamics simulations, this study investigates the stability of an NMR‐derived hexameric A*β*42 *β*‐barrel in aqueous solution, a fluid 1‐palmitoyl‐2‐oleoyl‐sn‐glycero‐3‐phosphocholine (POPC) bilayer, and a complex neuronal membrane. While the hexamer is unstable and conformationally heterogeneous in water, lipid environments provide essential structural reinforcement. Notably, the multicomponent neuronal membrane offers superior stabilization compared to POPC, supporting the *β*‐barrel in a stable transmembrane conformation. This superior stability is driven by the rigid scaffolding of the liquid‐ordered phase alongside specific electrostatic anchoring between the ethanolamine headgroups of the POPE component and the acidic A*β*42 residues E22/D23. We characterize a reciprocal relationship where the membrane stabilizes the *β*‐barrel architecture, while the peptide induces localized lipid disorder and flip‐flop translocation. Our findings demonstrate how membrane complexity and phase behavior dictate the stability of toxic A*β*42 oligomers, offering key insights into the membrane‐mediated mechanisms of neurotoxicity.

## Graphical abstract

Neuronal membrane order and the specific interplay of constituent lipids modulate the structural stability of A*β*(1–42) hexamers. This lipid environment facilitates the stabilization of a transmembrane *β*‐barrel conformation, providing a molecular basis for understanding how membrane composition influences the formation of stable amyloid pores in neurodegenerative pathways.

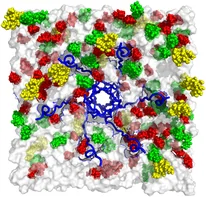

## 1 Introduction

**FIGURE 1:**
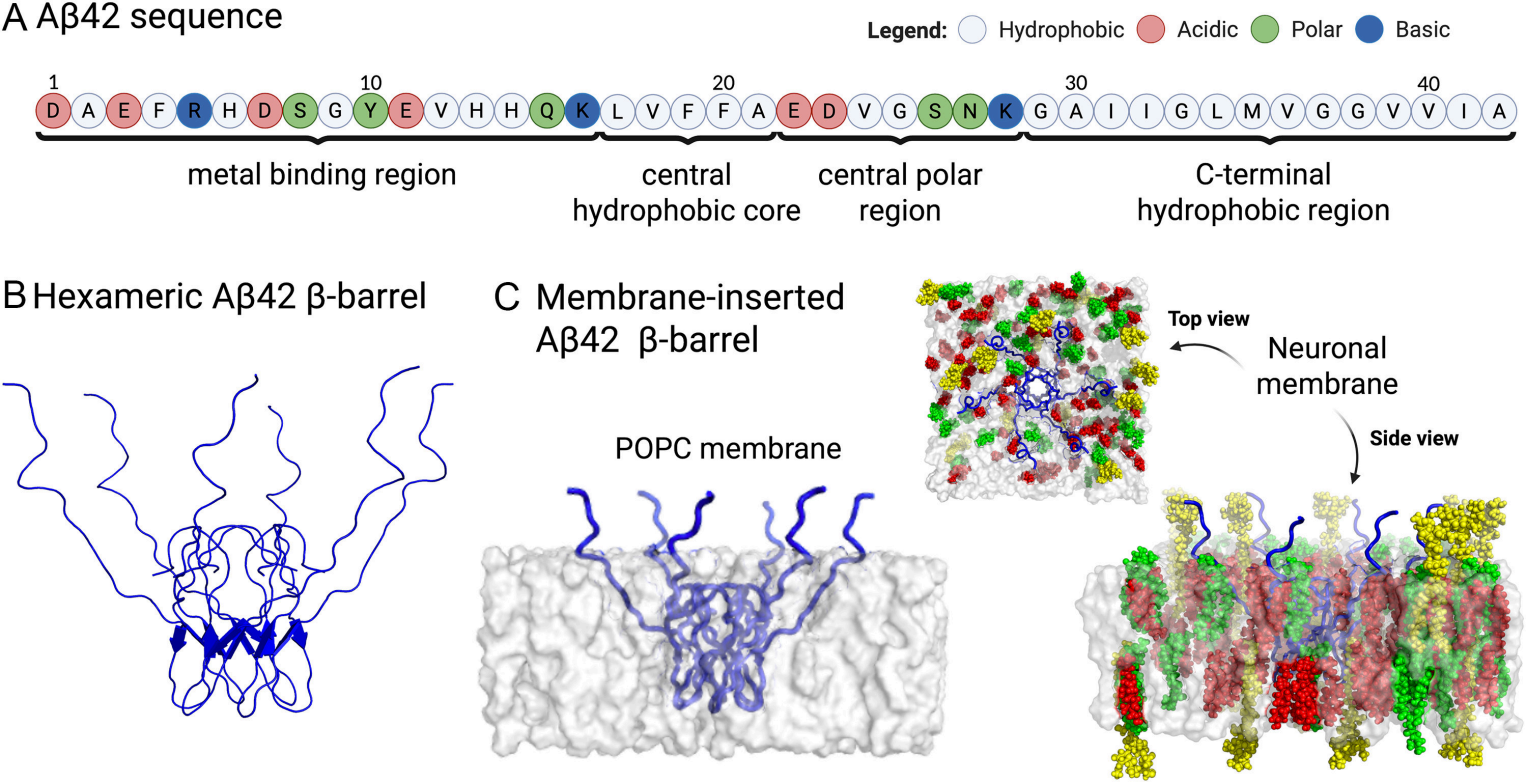
Sequence of A*β*42 and starting structures of the MD simulations. (A) The sequence of A*β*42, with different colors indicating the distinct physicochemical properties of the amino acids, according to the color key shown at the top. (B) As the starting structure for the A*β*42 hexamer, an NMR‐derived *β*‐barrel obtained for A*β*42_CC_ was used [1]. This structure was converted to the wild‐type sequence, and the missing N‐terminal residues 1–14 were added. The peptides are shown as cartoons. (C) The *β*‐barrel was simulated in solution (solvent not shown) and inserted into lipid membranes: a simple POPC membrane (shown as grey surface) and a neuronal membrane containing POPC, POPE, and POPS (collectively shown as a grey surface), Chol (red), SM (green), and GM1 (yellow). The *β*‐barrel was positioned such that the N‐terminal residues are located outside the membrane.

Amyloid‐*β* (A*β*) peptides, derived from the amyloid precursor protein via sequential proteolytic cleavage, play a significant role in the pathology of Alzheimer’s disease (AD). These peptides, particularly the 42‐residue form (A*β*42, see Figure 1A for the sequence), are known to aggregate and form insoluble fibrils, which are hallmark features of Alzheimer’s pathology [2, 3, 4]. The accumulation of A*β* in the brain is closely associated with neuroinflammation, synaptic dysfunction, and neuronal loss, leading to the cognitive decline observed in AD patients. A large body of studies suggests that the interaction of A*β* peptides with neuronal membranes may be a key contributor to their neurotoxic effects [5, 6, 7]. These interactions can disrupt membrane integrity and trigger toxic pathways, highlighting the importance of understanding the amyloid‐membrane dynamics in the context of AD.

The aggregation of A*β* peptides into fibrils progresses through a series of intermediate states, prominently involving the formation of oligomers with high structural adaptability [8], akin to a chameleon‐like behavior [9]. These soluble oligomers, typically ranging from small forms such as dimers and trimers to decamers, as well as larger aggregates like protofibrils, are increasingly recognized as the primary toxic species responsible for neuronal dysfunction and damage [10]. For small oligomers, toxicity increases with size: dimers are about three times more toxic than monomers, while trimers and tetramers are 8‐ and 13‐fold more toxic, respectively, correlating with higher *β*‐sheet content [11]. Notably, antiparallel *β*‐sheet oligomers, such as those stabilized through engineering a disulfide bridge into the A*β*42 peptide [1, 12], are exceptionally toxic, being 50 times more neurotoxic than fibrils or wild‐type A*β*42, which only forms transient oligomers [13]. A recent study analyzing soluble A*β* oligomers from eight brain regions revealed diverse sizes and structures, with smaller oligomers (~2 nm diameter, less than 100 nm in length) from hippocampal extracts showing the highest potency [14]. Their toxicity involves membrane disruption, calcium dysregulation, receptor blockade, and impaired neurotransmission, ultimately leading to synaptic dysfunction [15, 16, 17].

Studying the interactions between A*β* and neuronal membranes, or their mimics, is essential for understanding their mutual interplay and the implications for neurotoxicity. Neuronal membranes are primarily composed of a phospholipid bilayer enriched with cholesterol (Chol), glycoproteins, and various types of lipids, which contribute to their unique structural integrity [18]. This complex composition influences membrane dynamics and plays a crucial role in cellular signaling, making it critical to explore how A*β* disrupts these interactions and potentially contributes to neurodegenerative processes. Lipid metabolism is closely linked to Alzheimer’s pathology, characterized by disruptions in membrane structure [19]. Therefore, potential therapies should target lipids in addition to A*β* [19]. Research indicates that levels of phospholipids such as 1‍‍‐‍‍palmitoyl‐2‐oleoyl‐sn‐glycero‐3‐phosphocholine (POPC), 1‍‐‍palmitoyl‐2‐oleoyl‐sn‐glycero‐3‐phosphoethanolamine (POPE), and 1‐palmitoyl‐2‐oleoyl‐sn‐glycero‐3‐phospho‐L‐serine (POPS) decrease significantly in AD [6, 20].

Much focus has been placed on ganglioside lipids, particularly monosialotetrahexosylganglioside (GM1), to which A*β* preferentially binds when added to a lipid membrane [16, 21, 22, 23, 24, 25, 26]. However, conflicting results regarding the effect of gangliosides on A*β* aggregation have been reported; some studies indicate that they increase A*β* fibril formation [24, 27], while others suggest the opposite, even proposing neuroprotective properties [28]. A reduction in GM1 concentration in AD brains has been observed, leading to a higher aggregation propensity compared to other lipids [20]. High Chol levels promote A*β* formation by enhancing its production from the amyloid precursor protein [29]. Excessive Chol can alter membrane thickness and peptide orientation, affecting A*β*’s membrane insertion and aggregation pathway [30]. Moreover, membranes with diverse lipid compositions tend to resist fibril formation, indicating that lipid heterogeneity can protect against aggregation [31]. Many more such studies could be cited, but one of the main conclusions is that A*β*’s behavior depends significantly on the local membrane environment, where even slight changes in lipid composition can profoundly impact its toxicity and biological activity.

In this study, we utilize all‐atom molecular dynamics (MD) simulations to investigate the interaction between an A*β*42 hexamer and a complex neuronal membrane model. MD simulations have emerged as a robust and essential tool for studying amyloid systems [32], as they provide the atomistic resolution necessary to capture the transient structural transitions and specific lipid–peptide interactions that are often inaccessible to traditional experimental techniques [33]. The hexameric assembly studied here is a barrel‐like structure that serves as a building block for protofibril formation [1]. This architecture was determined by solid‐state NMR spectroscopy of the engineered A*β*42_CC_, which utilizes a disulfide bridge between A21C and A30C to stabilize the *β*‐hairpin motif formed by the central and C‐terminal hydrophobic regions (Figure 1B) [1]. To model the complex neuronal environment, we employed a multicomponent bilayer consisting of six lipid types—POPC, POPE, POPS, Chol, sphingomyelin (SM), and GM1—adopting a well‐characterized neuronal membrane model previously developed and validated in our group [25]. The hexamer was embedded within this membrane (Figure 1C) to assess its structural stability and its impact on membrane integrity. To provide a rigorous comparative framework, reference simulations were also conducted for the hexamer in aqueous solution and a pure POPC bilayer, as well as for the respective protein‐free membranes.

## 2 Methods

### 2.1 MD Simulations

#### 2.1.1 System Setup

Since the barrel‐like hexamer structure was obtained for A*β*42_CC_ [1], we reverted the cysteines at positions 21 and 30 to alanine. Additionally, the conformation of the first 14 N‐terminal residues of A*β*42 could not be resolved in the NMR experiments, so we added them using PyMOL. All simulation systems were subsequently prepared using CHARMM‐GUI [34], which included the creation of the lipid membranes and the insertion of A*β*42 into the lipid bilayers. As force fields, we employed CHARMM36 m for the peptides [35], the CHARMM36 lipid force field [36], and the CHARMM‐modified TIP3P water model [37]. Input and topology files were generated for running the MD simulation with GROMACS [38, 39] using version 2021.7.

For the neuronal membrane, a symmetric 10 nm × 10 nm lipid bilayer composed of 38% POPC, 24% POPE, 5% POPS, 20% Chol, 9% SM, and 4% GM1 was created (see Figure S1 for 2D structures of the lipids). Specifically, each leaflet comprises 76 POPC, 48 POPE, 10 POPS, 40 Chol, 18 SM, and 8 GM1 molecules, yielding a total of 400 lipids in the full bilayer. A pure POPC bilayer with the same size was also set up. In both bilayers, the A*β*42 hexamer was embedded such that residues 1–14 are positioned outside the membrane, while the *β*‐barrel‐like fold spans the membrane (Figure 1C). This configuration places hydrophobic residues 17‍–‍21 and 30–42 in the membrane core, while the charged residues K16, E22, D23, and K28 are located near the headgroup regions, consistent with their position in the amyloid precursor protein prior to cleavage [40]. Above and below the bilayer, water slabs with a thickness of 2.25 nm were added, incorporating a physiological salt concentration of 150 mM, thereby simultaneously neutralizing the overall systems.

Reference systems of both the POPC membrane and the neuronal membrane without the A*β*42 barrel were similarly generated. To characterize these membranes, we computed the lipid deuterium order parameters (*S*_CD_) and mass density profiles across the bilayer normal for the MD simulations of both setups (see Figures S2 and S3, respectively). For the POPC reference, the calculated *S*_CD_ values show excellent quantitative agreement with experimental ^2^H‐NMR measurements across both the *sn*−1 and *sn*−2 acyl chains, accurately reproducing the plateau values of the upper acyl chain segments [41, 42]. Additionally, while the phosphate peak‐to‐peak distance (∼4.0 nm) is slightly larger than experimental scattering estimates (∼3.7 nm [43]), this minor overestimation is a well‐documented characteristic of the CHARMM36 force field [36] and the profile successfully captures the expected fluid‐phase bilayer organization. While a low (9%) SM fraction might typically support a liquid‐disordered (*L*_d_) phase at 37 °C in simpler binary setups, the collective inclusion of 20% Chol and 4% GM1 gangliosides introduces a cooperative ordering effect. The large carbohydrate headgroups and saturated tails of GM1 pack tightly with Chol, systematically elevating the *S*_CD_ values and increasing the peak‐to‐peak headgroup distance in the mass density profiles relative to the pure fluid POPC control. These structural metrics confirm that our complex neuronal membrane successfully maintains a rigidified, liquid‐ordered (*L*_o_) matrix, whereas the pure POPC baseline reflects a characteristic *L*_d_ state.

#### 2.1.2 Membrane Simulations

First, energy minimization of the membrane systems, generated with CHARMM‐GUI, was performed using the steepest descent method until a maximum force of 1000 kJ/(mol⋅nm) on any atom was achieved. This was followed by five MD equilibration steps: a 125 ps simulation with a time step of 1 fs in an *NVT* ensemble (with *N* denoting the number of particles, *V* is the volume, and *T* is the temperature of the simulated system) utilizing a V‐rescale thermostat [44], a subsequent 125 ps simulation with a time step of 1 fs in an *NpT* ensemble (with *p* as the pressure in the simulated system) employing the V‐rescale thermostat and a C‐rescale barostat with semi‐isotropic pressure coupling [45], and three additional equilibration runs, each lasting 500 ps, using a 2 fs time step in an *NpT* ensemble with the V‐rescale thermostat [44] and the C‐rescale barostat [45]. The V‐rescale thermostat was configured with a coupling time constant of 0.1 ps for temperature coupling with 310.15 K as the target temperature, while the C‐rescale barostat employed a coupling time constant of 0.5 ps and a compressibility of 4.5 × 10^−5^ bar^−1^ to maintain pressure stability at 1 bar. In the first two equilibration runs, the peptide atoms were restrained in their positions, and these restraints were gradually lifted in the following three runs. Subsequently, production runs of 1 μs were conducted in an *NpT* ensemble, utilizing the V‐rescale thermostat, a C‐rescale barostat, and a time step of 2 fs. We used the LINCS algorithm [46] to set all‐bonds restraints during the equilibration runs, and lifted them to only apply to bonds involving hydrogen atoms during the production run. All MD simulations were performed under periodic boundary conditions. Nonbonded interactions calculated in real space were treated with a 1.2 nm cutoff for both Lennard‐Jones and Coulomb interactions. The particle mesh Ewald method [47], with a grid spacing of 0.1 nm for the fast Fourier transform, was applied for the calculation of Coulomb interactions. Trajectories were recorded every 20 ps for subsequent analysis.

#### 2.1.3 Simulation of the *Aβ*42 Barrel in Solution

An MD simulation of the A*β*42 hexamer in solution was conducted using a scheme similar to that of the membrane simulations, with the basic steps of energy minimization, equilibration, and the production run at 310.15 K and 1 bar remaining identical. Differences arose in the system preparation using CHARMM‐GUI, which automatically calculates the system and box size with a water thickness of 2.25 nm around the A*β*42 barrel. The final box size was 10 × 10 × 10 nm^3^. Second, in the absence of a membrane, only one equilibration run was performed in an *NVT* ensemble with a V‐rescale thermostat, lasting 125 ps with a time step of 1 fs, during which the peptides' movements were restricted. The subsequent production run was executed under the same conditions as in the production simulations of the membrane systems, with the exception that isotropic pressure coupling was applied.

### 2.2 Analyses

For the analysis of the MD simulations we applied GROMACS tools as well as Python packages, such as MDAnalysis [48, 49], MDtraj [50], and PyLipID [51]. For visualization of the analysis, we used the Python plotting library Matplotlib as well as Gnuplot [52]. PyMOL was used for molecular visualization and inspecting simulation trajectories [53].

#### 2.2.1 A*β*42 Barrel Stability

To assess the structural stability of the A*β*42 barrel, the root–mean‐square deviation (RMSD) of the C_α_ atoms was calculated for each production run relative to its initial starting structure. Additionally, we analyzed the stability of the individual A*β*42 peptides within the hexamer by calculating the root–mean‐square fluctuation (RMSF) of the C_α_ atoms per peptide, using the time‐averaged conformation of the corresponding A*β*42 chain as a reference. To identify the dominant A*β*42 hexamer structures from each simulation, we employed conformational clustering using the RMSD‐based algorithm implemented in GROMACS [54] with an RMSD cutoff of 0.6 nm to identify similar A*β*42 hexamer structures.

The structural stability is closely related to the secondary structure and the intra‐ and interpeptide interactions. Thus, we determined the time evolution of the secondary structure per peptide residue using the Define Secondary Structure of Proteins (DSSP) tool [55], which classifies the structure into helices (including *α*‐helix, *π*‐helix, and 3_10_‐helix), *β*‐sheets and *β*‐bridges, as well as bends, turns, and random coil. For simplicity, we consolidated these into three main classes: i) helices, ii) sheets, and iii) coils. The resulting data are visualized with the *x*‐axis representing simulation time, the *y*‐axis displaying the 42 amino acids of each of the six chains of the A*β*42 hexamer, and color indicating the three secondary structure classes. This analysis considered only MD conformations sampled every 0.1 ns.

The secondary structure is a direct consequence of hydrogen bonds (H─bonds) both within and between the peptides. To better understand the correlation between secondary structure and barrel stability, we utilized the *gmx hbond* tool from GROMACS to calculate the number of H─bonds within the A*β*42 hexamer, plotting their running averages over time. The criteria for identifying H─bonds included an angle of no more than 30° deviation from linearity for the angle formed by the H─bond donor atom (D), the hydrogen atom, and the H─bond acceptor atom (A), as well as a maximum D–A distance of 3.5 nm. To reveal stabilizing interactions within the hexamer, a contact matrix is calculated using a distance‐based method. For each pair of residues, the smallest Euclidean distance between any pair of atoms from the two residues is determined for each MD frame. A contact between two residues is recorded if this distance is less than 0.4 nm. Subsequently, a time‐averaged contact probability is calculated for each residue pair. The resulting matrix of intra‐ and interpeptide residue–residue contacts is then visualized as a heatmap.

#### 2.2.2 Membrane Properties and *A* *β*42–Membrane Interactions

To characterize the membrane properties, we calculated mass density profiles of the lipid phosphorus (P) atoms and the oxygen (O) atoms in the cases of GM1 and Chol using *gmx density*. The distances between density peaks provide an estimate of the average bilayer thickness per component. Additionally, we calculated the *S*_CD_ values by employing the Python script available at https://github.com/NMRLipids/MATCH [56]. Furthermore, to visualize the lateral distribution of specific membrane components, we employed the *gmx densmap* tool to generate time‐averaged spatial density maps, which reveal the local occupancy probabilities of specific particles. The 3D simulation box was partitioned into a regular grid, with atoms binned into cells for every trajectory frame. These counts were then normalized relative to the total number of frames and the grid cell volume. The final 2D plots represent a projection of this density, generated by integrating along the third dimension.

To evaluate whether the A*β*42 barrel influences membrane integrity, we quantified the membrane thickness as a measure of structural maintenance. Specifically, we calculated the local minimum thickness, defined as the shortest distance between the headgroups of the two bilayer leaflets (using P atoms for phospholipids or O atoms for Chol and GM1; Figure S1) across the trajectory. By focusing on minimum distances rather than global membrane averages, we can better capture the localized effects of A*β*42 on its immediate lipid environment. Furthermore, to evaluate the influence of the A*β*42 barrel on lipid acyl chain ordering, we computed the *S*_CD_ for lipids situated both within and beyond a 0.5 nm threshold of the peptide.

The PyLipID tool was utilized to analyze peptide–lipid interactions [51]. It employs a distance‐based contact definition to determine, for each simulation frame, whether a lipid atom is within the specified cutoff distance of 0.5 nm of a peptide residue. To identify lipid‐binding sites on the peptides, residues that consistently form contacts with lipids are clustered. Initially, these residues are grouped based on their contacts. The density‐based spatial clustering of applications with noise algorithm is then applied to extract groups of residues that form a stable interaction surface [57], which are referred to as binding sites. Geometric clustering can further be employed to assess the typical lipid binding pose, and the binding sites can be characterized by their frequency and residence time. The peptide–lipid interactions were further analyzed by calculating their interaction energies using the potential energy function of the force field, distinguishing between Coulomb and Lennard‐Jones interactions. To obtain these energies, the necessary .ndx and .mdp files were created to define the relevant energy groups, and the calculations were carried out using the *gmx rerun* function of GROMACS [38]. The interaction energies are presented as time averages, accompanied by their minimum and maximum values.

## 3 Results

### 3.1 Structural Stability of the *Aβ*42 Barrel

**FIGURE 2:**
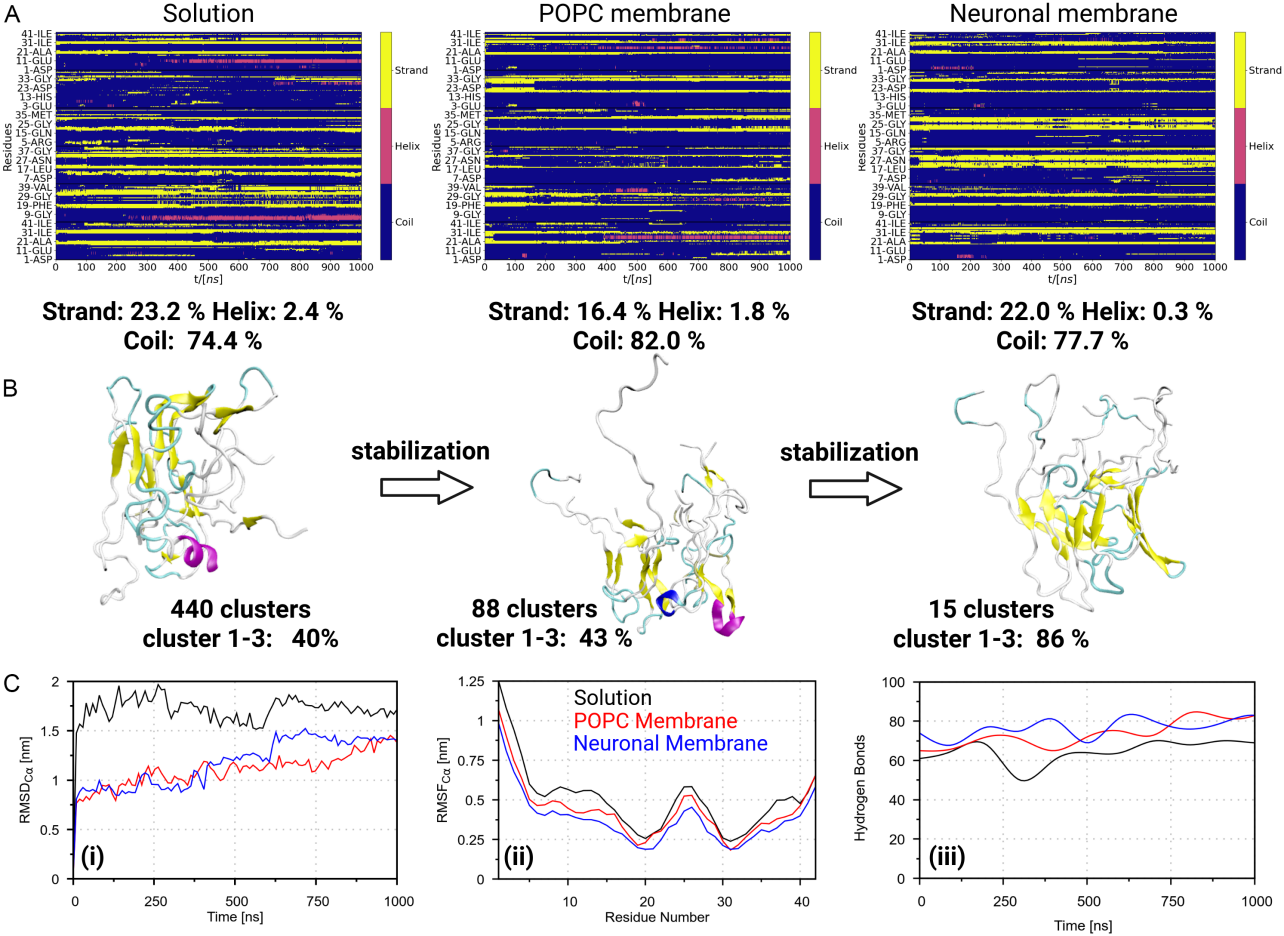
Structural stability and fluctuations of the A*β*_42_ *β*‐barrel across different environments. (A) Evolution of the secondary structure during the MD simulations. The secondary structure per residue for all six A*β*_42_ peptides is color‐coded: yellow for *β*‐strands, pink for helices, and blue for coils. The *y*‐axis represents the concatenated sequence from residue 1 of chain 1 to residue 42 of chain 6. Results are compared for the *β*‐barrel in aqueous solution (left), a POPC membrane (middle), and a complex neuronal membrane (right). Values below the plots represent the time‐averaged percentages of secondary structure (*β*‐strand, helical, and random coil elements) calculated across the entire trajectory. (B) Representative structures of the most populated clusters obtained from structural clustering (displayed as cartoons). Color scheme: *β*‐sheets (yellow), helices (pink), turns (cyan), and coils (white). The total number of clusters and the cumulative population of the top three clusters are provided for each system. (C) Quantitative structural metrics: (i) time evolution of the chain‐averaged C_α_‐RMSD; (ii) chain‐ and time‐averaged C_α_‐RMSF per residue; and (iii) evolution of the total number of hydrogen bonds (intra‐ and inter‐peptide) within the *β*‐barrel. Data are shown for the simulations in solution (black), in a POPC membrane (red), and in a neuronal membrane (blue).

We begin the analysis of the MD trajectories by assessing the stability of the A*β*42 barrel structure, considering both the overall stability of the hexameric assembly and the maintenance of the individual structure for each A*β*42 peptide. To evaluate the latter, we determined the time evolution of the secondary structure for each A*β*42 residue using DSSP (Figure 2A). The results indicate that the peptides maintain their *β*‐hairpin structure most effectively in the neuronal membrane, with partial local stabilization occurring in solution, while the POPC membrane supports the *β*‐hairpin structure the least. In solution, various often short‐lived *β*‐structures are observed, along with transient helices, reflecting a competition between folding motifs. Notably, monomeric A*β*42 in solution hardly adopts helical structures [58, 59], and when it does, it exists in a high‐energy state [60]. However, the formation of helical structures in A*β*42 oligomers has been previously postulated [61] and shown to be energetically accessible upon interactions with hydrophobic surfaces [62]. In the POPC membrane, the fraction of *β*‐structures decreases (from 23.2% in solution to 16.4% in the membrane), and, similar to the solution environment, intermittent helices appear. In the neuronal membrane, however, the *β*‐hairpins characteristic of the hexameric barrel remain stable (22.0% *β*‐sheet content), and helices do not form, apart from short‐lived turns that are classified as helices by DSSP. The secondary‐structure percentages across the full trajectories in each environment confirm this.

The analysis of the time‐averaged intramolecular contacts (Figure S4) supports similar conclusions regarding the stability of the peptide structures. For instance, in peptide chain 5 of A*β*42 in solution, the contacts indicative of the *β*‐hairpin vanished completely, whereas formation of helices can be observed in the N‐terminal halves of chains 2 and 6. In the POPC membrane, the *β*‐hairpin signature became generally weaker and shorter, particularly in chain 3, while it remained strong and distinct for all six peptides within the A*β*42 barrel immersed in the neuronal membrane.

To obtain an overview of the hexamer structures and their variability, we applied conformational clustering. The representative structures of the most populated cluster for each system are visualized in Figure 2B. In solution, 440 clusters were detected, with the three largest clusters accounting for only 40.2% of the total structures, indicating that the barrel structure is not stable in this environment. It is important to note that this *β*‐barrel structure has been identified as a building block of A*β* fibrils [1], suggesting that the barrel requires additional structural support to maintain stability. While neighboring A*β* barrels can provide this support in protofibrils, a lipid membrane environment also contributes; in the POPC membrane, the number of identified clusters decreases to 88, although the three largest clusters still represent only 43.0% of the sampled conformations. Considerably greater stabilization is achieved in the neuronal membrane, where only 15 clusters were sampled, with the first three clusters collectively comprising 85.6% of the conformations. Under all three conditions, the representative structures display *β*‐strand‐rich hexamers, but with the presence of helices and a loss of barrel geometry in solution and the POPC membrane. Only in the neuronal membrane does the barrel‐like geometry remain visible. These observations align with the varying stabilities of the *β*‐hairpins identified from the secondary structure analysis.

We complete the analysis of structural stability by comparing global and local flexibility, as represented by RMSD and RMSF, as well as the number of H─bonds between the peptide chains. The RMSD relative to the initial barrel structure (Figure 2Ci) confirms that the membrane environments stabilize the A*β*42 hexamer compared to when it is in solution. In the latter case, the RMSD quickly rises above 1.5 nm and fluctuates between 1.0 and 1.5 nm. In both membrane environments, the RMSD increases gradually and remains below 1.5 nm. Since RMSD alone does not provide insights into local flexibilities, we include the RMSF values for a more comprehensive view (Figure 2Cii). This analysis reveals that two regions exhibit increased mobility across all systems: the N‐terminal residues 1–10 and the turn region between residues 20 and 28. The increased flexibility in the N‐terminal region can be attributed to its intrinsic disorder based on its amino acid composition [9], which also explains its absence from the NMR‐derived structure of the *β*‐barrel [1]. The turn region connects the two *β*‐strands involving residues 17–21 and 31–42 and shows in solution and in the POPC membrane a tendency toward helix formation, as evidenced by the cluster structure for the POPC system in Figure 2B. The RMSF profiles indicate that the A*β*42 hexamer is most mobile in solution and most stable in the neuronal membrane. In the latter, it is particularly the N‐terminal region of peptide chain 6 and the turn region of chain 4 that exhibit greater dynamics compared to the other four chains (Figure S5). Without these regions, the difference in RMSF between the neuronal system and both the solution and POPC systems would be even more pronounced.

Finally, analysis of the hydrogen bond count within the A*β*42 hexamer (Figure 2Ciii) reinforces that the *β*‐barrel is less stable in solution, featuring fewer than 70 intra‐ and interpeptide H─bonds. In contrast, this number fluctuates between 75 and 85 in the two membrane environments, with the hexamer in the neuronal membrane generally exhibiting the highest occupancy. This disparity arises because intra‐ and interpeptide H─bonds in water must compete with H─bonds formed between A*β*42 and the solvent; such competition is absent within the hydrophobic membrane cores, which lack polar hydrogen atoms.

### 3.2 A*β*42 Barrel–Membrane Interactions

#### 3.2.1 Density Maps and Lipid Binding Sites

To understand how the membrane systems stabilize the hexameric A*β*42 *β*‐barrel, we assessed the interactions between the lipids and the hexamer. For this purpose, density maps of the different molecules (peptides, water, and lipids) were calculated, and the binding sites of the lipids on the A*β*42 peptides were analyzed.

**FIGURE 3:**
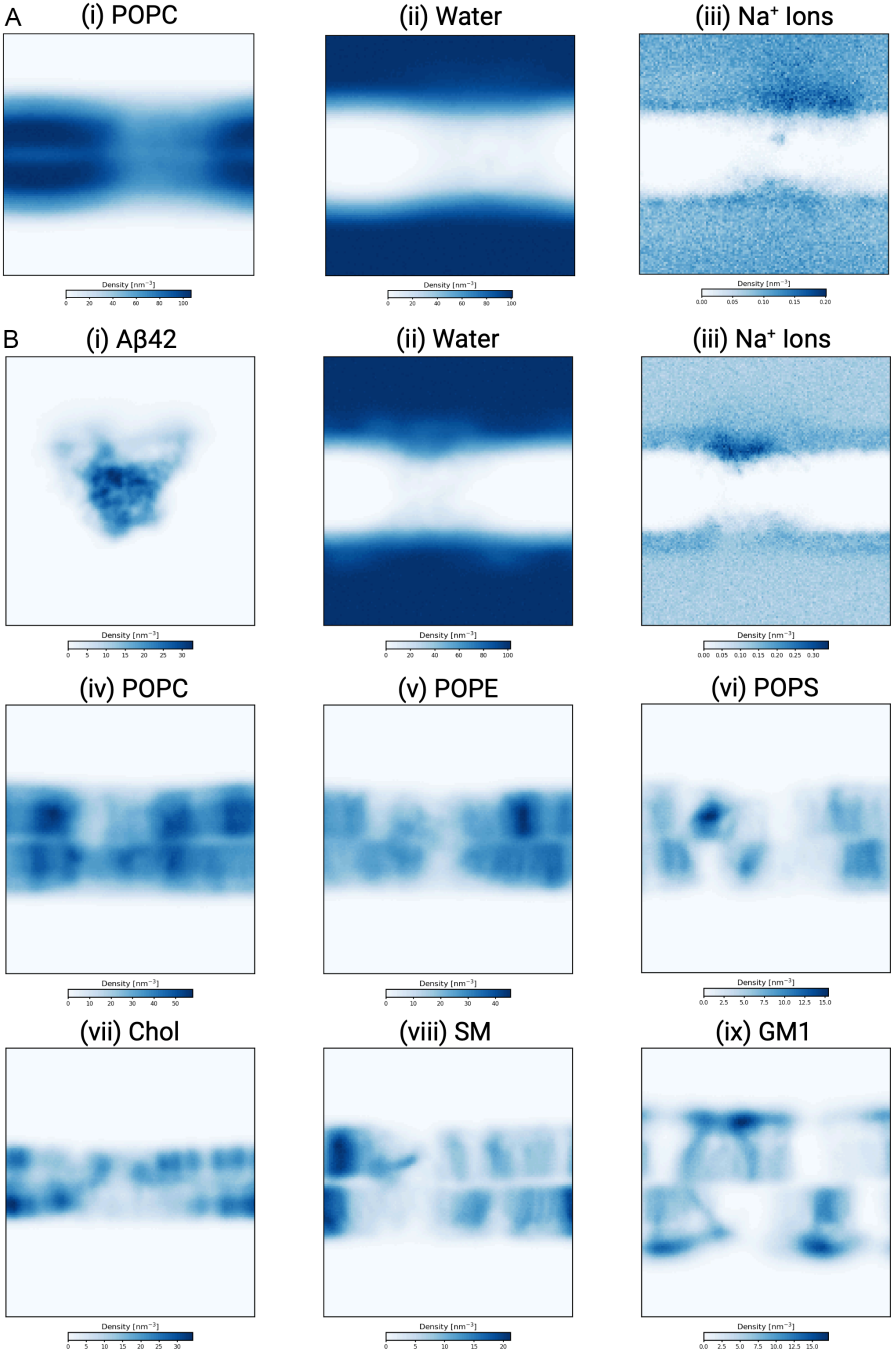
Component‐specific density maps for the A*β*_42_ *β*‐barrel in POPC and neuronal membranes. (A) Density maps calculated from MD simulations of the *β*‐barrel in a POPC membrane. Labels above each panel specify the respective system component. The corresponding density map for the *β*‐barrel is provided in Figure S4A. (B) Density maps for the *β*‐barrel simulated in a neuronal membrane, with components indicated by the labels above each panel. Densities are presented as heatmaps (nm^−3^), with corresponding color scales provided below each panel.

In the pure POPC bilayer, the only interaction available for A*β*42 is with POPC, leading to a dense distribution of POPC around the *β*‐barrel, as indicated by the dark blue color in the density map in Figure 3Ai. Only at the location where the A*β*42 hexamer resides (Figure S6A), the POPC density is reduced. The POPC binding sites on the peptides are distributed throughout the transmembrane region of the barrel, yet some of the N‐terminal residues also form contacts with POPC, due to interactions with the headgroups on the exterior side of the membrane (Figure S6B). The density map of the A*β*42 barrel in the POPC membrane (Figure S6A) indicates that it spans the membrane and is narrower in the lower leaflet than in the upper leaflet. Water molecules can enter the membrane core within the barrel, which is visible in the water density map as a faint blue color in the membrane region (Figure 3Aii). The Na^+^ density map reveals that these ions accumulate at the headgroup regions of both membrane leaflets, with the highest density observed at the entrance of the A*β*42 barrel in the upper leaflet, while some sodium ions migrate into the membrane core (Figure 3Aiii). A closer inspection further reveals that water and Na^+^ can diffuse anywhere, i.e., independent of A*β*42, through the membrane. This confirms the characterization of POPC as a lipid that forms liquid‐disordered membranes, which are intrinsically permeable to the passive diffusion of small solutes [63].

For the A*β*42 barrel in the neuronal membrane, slightly different observations are made regarding the water and Na^+^ densities, as illustrated in Figure 3Bi–iii. One notable difference is that the membrane‐inserted A*β*42 hexamer facilitates greater entry of water and ions into the membrane core, which results from the higher stability of the *β*‐barrel in the neuronal membrane, maintaining a more pore‐like geometry compared to the POPC membrane. Conversely, the neuronal membrane presents a higher barrier to the random diffusion of water and Na^+^ into the membrane, which is due to the more organized matrix structure in the neuronal membrane [25]. Chol and SM are known for their role in increasing lipid order, causing the neuronal membrane to be in the liquid‐ordered state. In another work, we demonstrated that Chol is a crucial membrane component for preventing water ingress into lipid membranes and for maintaining membrane integrity, even under oxidative conditions [64].

Regarding the distribution of the lipids within the neuronal membrane, most are unevenly distributed. The two most prevalent lipids, POPC and POPE, which together account for 62% of the membrane, exhibit the most even distributions; however, some enrichments are noted, particularly in the upper leaflet (see Figure 3Biv/v). Chol also displays a relatively uniform distribution, whereas POPS, SM, and GM1—each contributing less than 10% to the membrane—form distinct clusters. This aligns with findings from the pure neuronal membrane, where notable clustering was observed for GM1 and SM, while the other lipids did not tend to self‐associate, and preferred mixed pairs were not identified [25]. The clustering of GM1 is driven by interactions between its oligosaccharide headgroups, which extend outward from the membrane surface. In contrast, SM clustering is facilitated by an extensive hydrogen‐bonding network involving the amide N–H and hydroxyl groups (Figure S1). These groups serve as both H─bond donors and acceptors, which not only increases the intrinsic rigidity of SM lipids but also contributes to the overall stiffness of the neuronal membrane.

#### 3.2.2 Lipid Binding Sites

To characterize the lipid–binding interactions, PyLipID [51] was used to identify specific binding sites on the A*β*_42_ peptides. In the pure POPC membrane, a dense distribution of POPC around the A*β*42 hexamer is observed (Figure S6B), reflecting its role as the sole available interaction partner. In the neuronal membrane, however, interactions with POPC are markedly depleted and are instead replaced by interactions with the other lipid components (Figure S5). These preferences are largely driven by the specific locations of the binding sites; for instance, GM1 preferentially interacts with N‐terminal residues via its protruding oligosaccharide headgroups. Further insights are obtained from the lipid residence times at individual A*β*_42_ residues. While these data are presented only for the most populated binding sites, we supplement this temporal data with an analysis of interaction energies between residues and the respective lipids (irrespective of the binding site) to better assess overall affinity.

In the pure POPC bilayer, the longest residence times are observed at the N‐terminal residues Y10–Q15 and F19–A21 (Figure S6C). However, the interaction energy analysis reveals that POPC contacts are distributed across the entire A*β*42 sequence (Figure S6D). These interactions are governed by both Coulombic and van der Waals (vdW) forces. The N‐terminal residues, which primarily interface with the POPC headgroups, are dominated by electrostatic contributions, though they also maintain hydrophobic character at positions such as L17 and V18. Conversely, within the hydrophobic core of the membrane, vdW interactions serve as the primary stabilizing force, particularly for the C‐terminal residues I31–L34. It is important to note that hydrophobic interactions, as modeled by the vdW potential, only partially capture the full hydrophobic effect, an entropic phenomenon related to water entropy. Nonetheless, using vdW energies as a surrogate is a common practice to gauge the driving forces behind protein interactions by estimating the shielding of hydrophobic amino acids from water. Overall, the analysis of the peptide–POPC interactions for the A*β*42 hexamer in the pure POPC membrane indicates a predominance of rather nonspecific interactions, which are insufficient to maintain the shape of the *β*‐barrel, at least on the simulated time scale.

**FIGURE 4:**
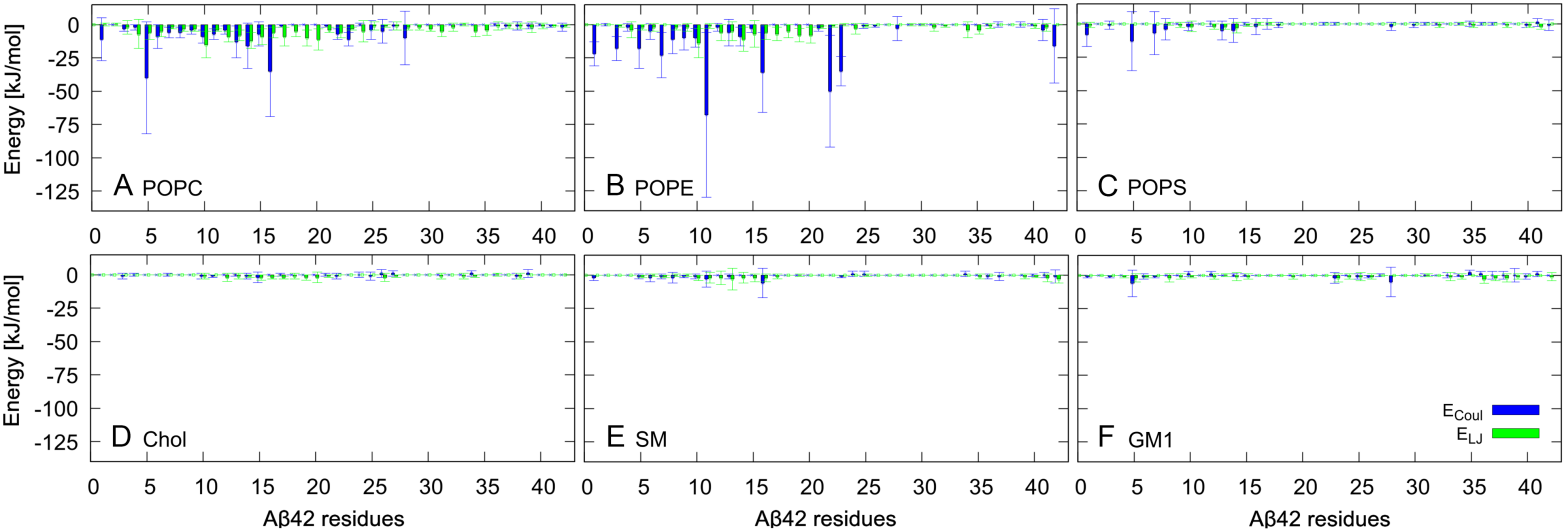
Interaction energies between A*β*42 residues and neuronal membrane lipid components. Energies are time‐ and chain‐averaged, with fluctuations represented by the minimum and maximum values. Interactions are decomposed into electrostatic (blue) and Lennard‐Jones (green) components. Increasingly negative values denote stronger attractive interactions. Results are shown for the interactions of A*β*42 with (A) POPC (38 mol%), (B) POPE (24 mol%), (C) POPS (5 mol%), (D) Chol (20 mol%), (E) SM (9 mol%), and (F) GM1 (4 mol%). Equivalent data for the A*β*42–POPC interactions in the pure POPC membrane system are provided in Figure S4D.

**FIGURE 5:**
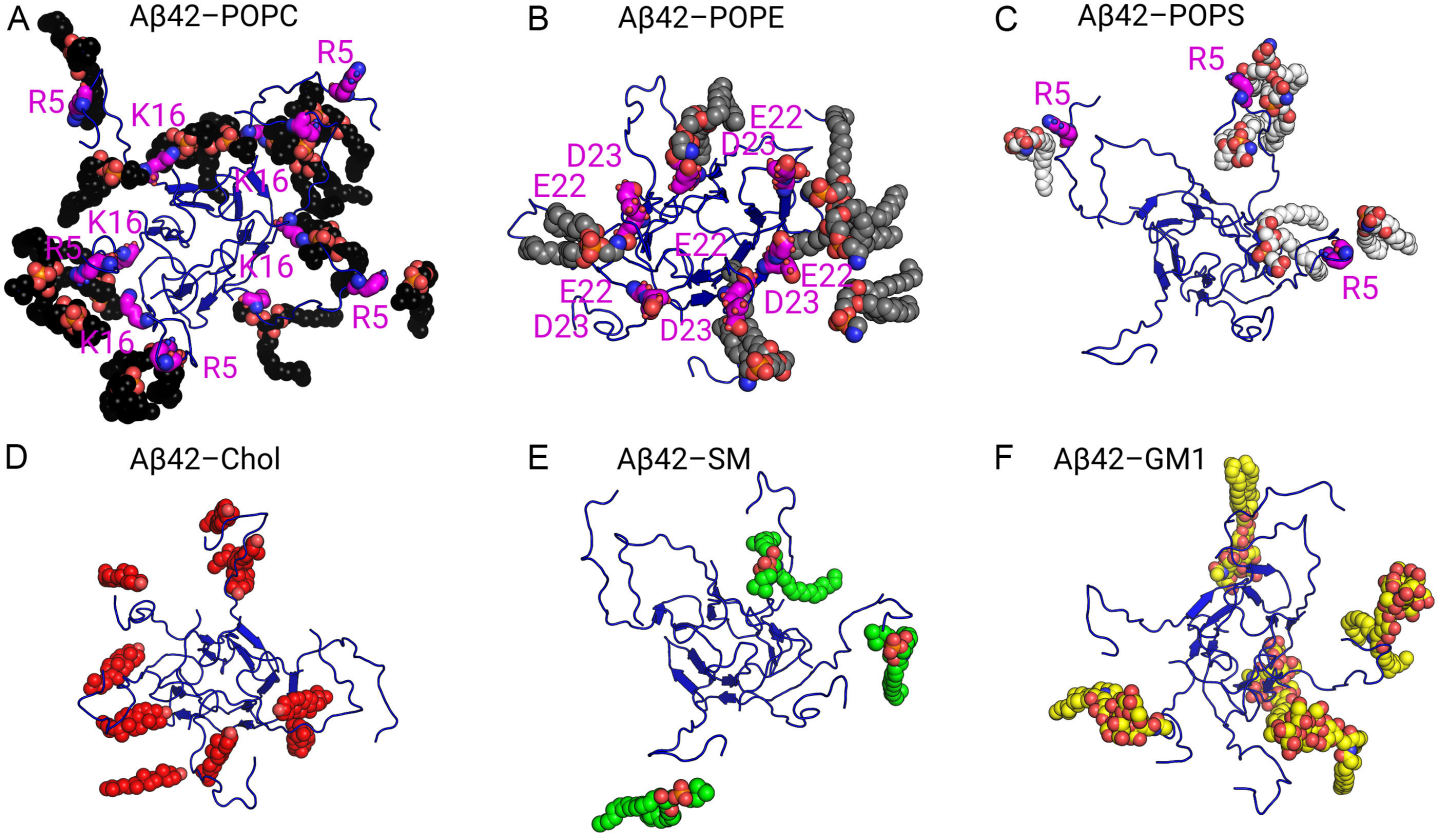
Structural representation of key interactions between A*β*42 residues and neuronal membrane components. The *β*‐barrel is shown as a blue cartoon, with side chains of the most relevant interacting residues highlighted in magenta (CPK representation; N atoms in blue, O atoms in red). Panels A–C show specific residue‐level interactions with (A) POPC, (B) POPE, and (C) POPS. For (D) Chol, (E) SM, and (F) GM1, representative snapshots of the local lipid environment are shown; individual residues are not highlighted in these panels as no specific, dominant residue‐wise binding hotspots were identified. All lipids are shown in CPK representation (H atoms omitted for clarity), with lipid tails following the color code in Figure 1, P atoms in orange, N in blue, and O in red.

In the neuronal membrane, different binding modes between A*β*42 and the various lipid species are observed (Figure S7). Based on residence times for the most populated lipid binding mode, POPC binds stably to residues H13, Q15, and F19, followed by E11 and H14; for the former three residues, the residence time is 1 *μ*s, indicating that the bound POPC molecule remains unchanged during this time. These interactions occur in and near the headgroup region of POPC in the upper leaflet, which is supported by the dominating electrostatic energies governing the interactions between the peptides and POPC (Figure 4A). The highest interaction energies are observed for R5 and K16 (Figure 5A). K16 is situated within a local environment where neighboring residues exhibit the highest residence times for POPC. In contrast, POPS, with its overall negative charge, shows no preference for the negatively charged A*β*42 peptides (−3 charge). Based on residence times, only H14 maintains a stable contact in the dominating POPS binding mode; however, this interaction is weak, as indicated by the interaction energy analysis. Still, some low energetic interactions with R5 were found, implying interactions with the headgroup (Figure 5C). The lipid molecule with the most significant and strongest interactions with A*β*42 is POPE. This is evidenced by the broad distribution of residue–lipid contacts of considerable duration across the entire peptide sequence (Figure S7), particularly the strong electrostatic interactions involving residues D1, E3, R5, E11, K16, E22, and D23 (Figure 4B). The interactions with negatively charged residues are facilitated by the ${\text{NH}}_{3}^{+}$ groups in POPE, instead of the bulkier $N{\left(\mathrm{CH}3\right)}_{3}^{+}$ groups in POPC, making such interactions less likely for POPC. The interactions of POPE with E22/D23 are especially relevant, as they stabilize the membrane‐spanning *β*‐barrel structure by providing an anchoring point in the headgroup region of the lower leaflet (Figure 5B).

With regard to the other three membrane constituents, Chol, SM, and GM1, their interactions with A*β*42 are weaker both in terms of duration (Figure S7) and energy (Figure 4). None of the three exhibit noteworthy electrostatic interactions. Although the hydrophobic interactions are generally weaker, they are also less relevant compared to those observed for POPC and POPE. The primary structural distinction between POPC and SM lies in the replacement of the glycerol backbone with a sphingosine moiety. The latter introduces an amide group, a free hydroxyl group at the C3 position, and a trans–double bond between C4 and C5. As previously discussed, the amide N–H and hydroxyl groups enable the formation of an extensive inter‐lipid hydrogen‐bonding network. This network enhances the packing density and rigidity of SM domains, making them less energetically favorable for A*β*42 intercalation (Figure 5E). In contrast, the ester groups in POPC function solely as H─bond acceptors. Lacking internal H─bond donors, POPC remains in a more fluid and accessible state, which facilitates more frequent and stable interactions with the A*β*42 hexamer. This aligns with our previous findings, where we systematically studied the effects of increasing SM in a POPC‐based bilayer on the interactions with an externally added single A*β*42 peptide [65]. A*β*42 was able to embed its N‐terminus in the POPC bilayer, while the rigidity of the SM‐enriched bilayer prevented embedding but promoted intrapeptide interactions. In this context, SM can be regarded as a stabilizing factor for the *β*‐barrel, as it strengthens both intra‐ and interpeptide hydrogen bonds.

The interaction energies between Chol and GM1 with A*β*42 are negligible; however, their contact times indicate that interactions do occur. Chol serves as a membrane component that supports the *β*‐barrel by shielding the hydrophobic residues of the *β*‐hairpins, particularly given that Chol generally enhances the structural integrity of membranes (Figure 5D). For GM1, we observe that interactions primarily occur through its oligosaccharide headgroups with the N‐terminal residues (Figure 5F). Our previous study [25] already highlighted the significant role of GM1 headgroups during the early stages of extracellular docking and capturing of the amyloid peptide. However, once the peptide becomes embedded in the membrane, GM1’s role in peptide stabilization changes. Since the lipid backbone and tails are identical to those of SM, GM1 also contributes to the stability of the transmembrane *β*‐barrel by promoting intra‐ and interpeptide interactions rather than interactions with the lipid tails.

#### 3.2.3 Effects on the Membrane

We conclude our analysis by quantifying additional effects of the A*β*42 hexamer on the lipids and membrane, beyond the previously discussed entry of water and Na^+^ ions into the membranes. As established earlier via phosphorus mass density profiles (Figure S3), the *average* headgroup‐to‐headgroup distances of our peptide‐free models align closely with experimental literature, confirming the structural validity of our membrane setups. These density profiles also confirmed the expected phase states: the pure POPC bilayer adopts a more fluid *L*_d_ phase, whereas the complex neuronal membrane exhibits a thicker *L*_o_ phase. This increased fluidity in the pure POPC membrane partly explains why it provides less structural support to the A*β*42 *β*‐barrel compared to the more rigid neuronal membrane.

**FIGURE 6:**
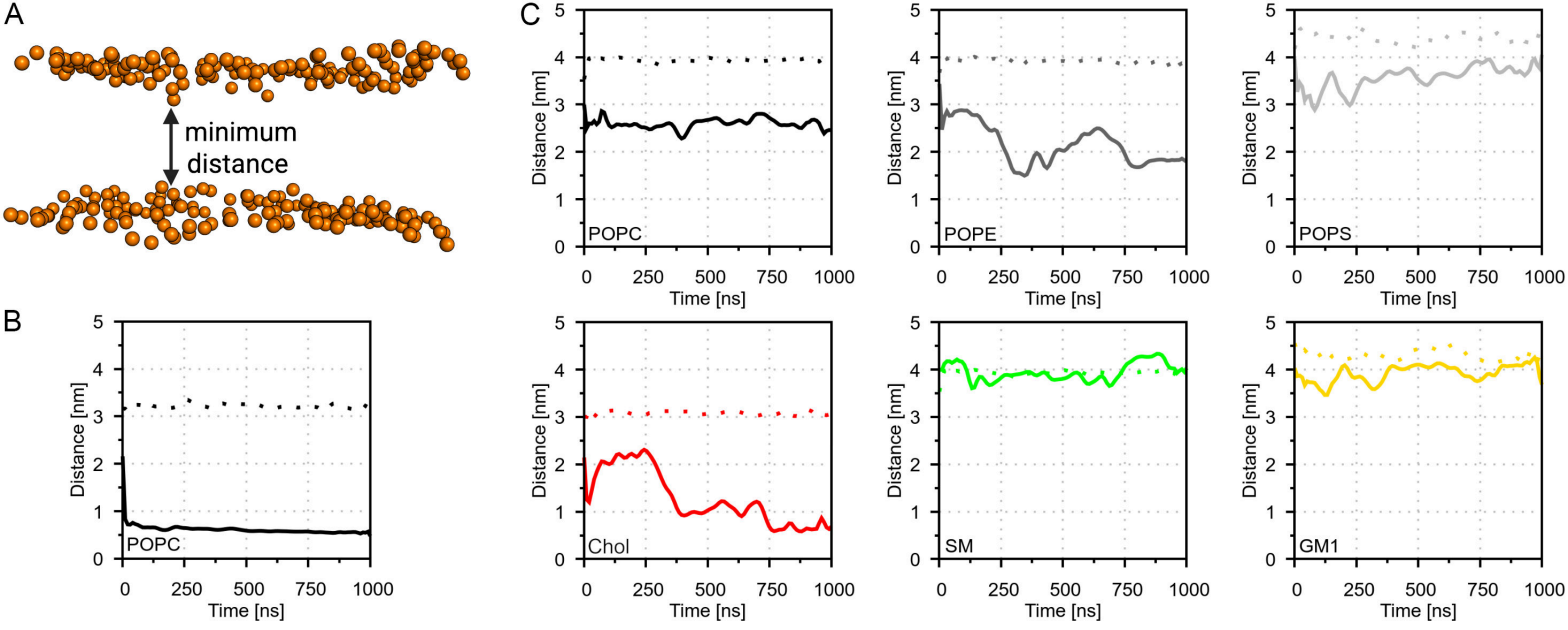
Membrane thickness reduction induced by the A*β*42 *β*‐barrel. (A) Schematic of the distance measurement between the phosphorus (P) atoms (or oxygen (O) atoms for Chol and GM1; see Figure S1) of the upper and lower leaflets. This minimum headgroup‐to‐headgroup distance serves as a proxy for local membrane thickness. (B) Comparison of minimum headgroup‐to‐headgroup distances in the POPC membrane with (solid) and without (dotted) the *β*‐barrel. The significant decrease to ~0.5 nm in the presence of the *β*‐barrel indicates a lipid flip‐flop event. (C) Minimum headgroup‐to‐headgroup distances in the neuronal membrane for all six lipid species (solid lines, color‐coded as in Figure 1) compared to the equilibrium distances in a protein‐free neuronal membrane (dotted lines).

Having established the global properties of the bulk membrane, we then focused on the highly localized perturbations in the immediate vicinity of the *β*‐barrel. To capture these individual lipid reorientations, we quantified the *minimum* distance between lipid headgroups across the two leaflets, rather than relying on the average bilayer thickness. As Figure 6 shows, in the absence of A*β*42, this minimal distance remains relatively stable, fluctuating around 3.2 nm for the pure POPC membrane, ~4.0–4.5 nm for the phospholipids and sphingolipids in the neuronal membrane, and 3.0 nm for Chol. Against this unperturbed background, the A*β*42 *β*‐barrel introduces a geometric mismatch, as it is too short to comfortably span the membrane core (see Figure 1C). Consequently, to satisfy hydrophobic matching, the specific lipids that interact most strongly with the *β*‐barrel are physically drawn toward the membrane interior. This mechanism explains the drastic, localized reductions in the minimum distance between their headgroups in the presence of the peptide, which should not be misinterpreted as a global thinning of the membrane. To further differentiate between these localized boundary effects and the global membrane state, we computed the *S*_CD_ for lipid acyl chains situated within and beyond a 0.5 nm threshold of A*β*42 (Figure S2). For lipids beyond 0.5 nm, the *S*_CD_ values closely mirror those of the peptide‐free membrane, confirming that the structural integrity of the bulk membrane is maintained and that perturbations are strictly confined to the peptide’s annular shell.

Within the 0.5 nm interacting shell around A*β*42, the phospholipids POPC, POPE, and POPS exhibit distinct behaviors. POPE experiences the most extreme deformation; its minimum headgroup distance decreases to under 2 nm with substantial variance, driven by extensive interactions with A*β*42, specifically involving the E22/D23 cluster in the lower leaflet. POPC maintains a reduced yet stable headgroup distance of ~2.5 nm with low fluctuations, suggesting it adopts an optimal configuration for interactions with the *β*‐barrel. In contrast, POPS shows the least change among the phospholipids: after an initial drop, the distance recovers by the end of the simulation to closely match the peptide‐free neuronal membrane. This indicates that A*β*42 and POPS tend to avoid each other, a finding further corroborated by the *S*_CD_ analysis, which reveals that the POPS palmitoyl chain is less perturbed than those of POPC and POPE.

Among the sphingolipids, the headgroup spacing of SM and GM1 remains the least affected by the physical pull of the peptide. For GM1, we observe only a marginal compression (shifting to the 3.5–4.3 nm range), and its *S*_CD_ values remain completely unaffected by the presence of A*β*42, confirming minimal overall perturbation. SM similarly retains its initial headgroup‐to‐headgroup distance. However, unlike GM1, the *S*_CD_ analysis reveals a distinct decrease in SM lipid acyl chain order within the 0.5 nm shell. This indicates a decoupled response: while the SM headgroups remain stably anchored at the membrane‐water interface, their hydrophobic tails are significantly disordered by the adjacent hydrophobic mismatch of the short β‐barrel. Finally, the minimum distance between Chol oxygen atoms fluctuates initially around 2.0 and 1.0 nm before settling at almost 0.5 nm, indicative of flip‐flopping behavior.

For the pure POPC membrane with the A*β*42 hexamer included, the highly localized *minimum* distance between headgroups was drastically reduced to almost 0.5 nm (Figure 6B). This extreme spatial disruption is driven by a profound loss of lipid tail order: analysis of the adjacent POPC lipids reveals that their *S*_CD_ values plummet to below ~0.1 for all carbon atoms across both acyl chains (Figure S2). Stripped of their structural rigidity by the *β*‐barrel, these highly disordered lipids undergo flip‐flopping between leaflets, similar to the behavior observed for Chol in the neuronal membrane. Due to the static nature of GROMACS index groups, lipids that relocate to the opposing leaflet during the simulation remain assigned to their initial leaflet. In such cases, the reported 0.5 nm minimal distance no longer represents a localized collapse of the bilayer thickness, but instead reflects the proximity between headgroups that have moved into the same leaflet. Nonetheless, it effectively serves as geometric confirmation that A*β*42‐induced POPC flip‐flopping has occurred. Furthermore, these findings indicate that the impact of the A*β*42 *β*‐barrel on the POPC component is significantly attenuated in the neuronal membrane. This is due to the membrane‐stabilizing effects of Chol, SM, and GM1, which promote a membrane transition toward the more rigid *L*_o_ phase.

## 4 Discussion and Conclusion

This study utilized atomistic MD simulations to investigate the behavior of a hexameric A*β*42 *β*‐barrel across three distinct environments: aqueous solution, a pure POPC bilayer, and a complex neuronal membrane. We characterized the structural stability, peptide–lipid interactions, and the reciprocal effects of the peptides on membrane integrity to elucidate the molecular mechanisms governing barrel stabilization.

**FIGURE 7:**
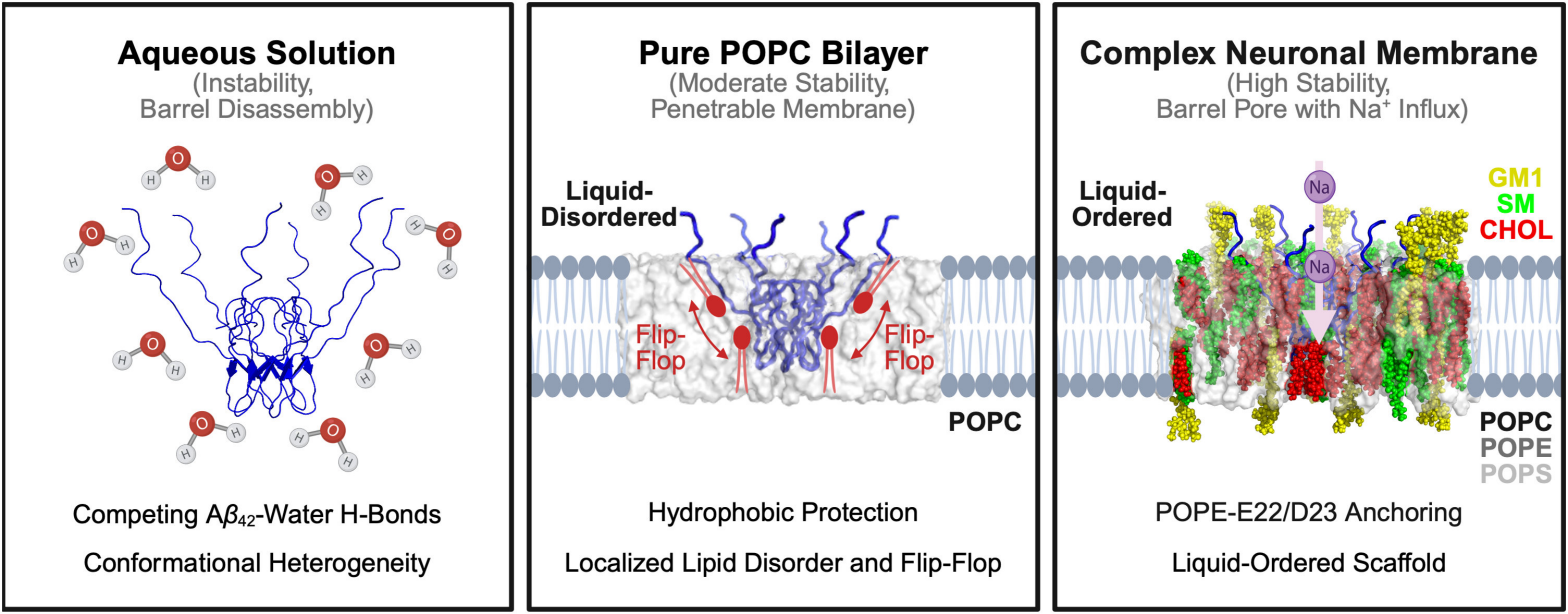
Summary of A*β*42 hexamer behavior in solution and membrane environments. Schematic representation of the A*β*42 *β*‐barrel in (left) aqueous solution, where the oligomer is unstable and fails to maintain a pore structure, (middle) a simple, fluid POPC membrane, characterized by structural instabilities, severe localized lipid disordering, and membrane thinning, and (right) a complex neuronal membrane mimic, where the liquid‐ordered (*L*_o_) phase and specific lipid–peptide interactions cooperatively stabilize the membrane‐inserted barrel.

A central finding is that the stability of the A*β*42 *β*‐barrel is highly environment‐dependent (Figure 7). In aqueous solution, the barrel is inherently unstable; the hexamer remains highly dynamic and samples a heterogeneous conformational landscape, reflecting the intrinsically disordered nature of the A*β*42 monomer [9]. While the POPC membrane provides moderate stabilization and leads to a more compact global structure, the neuronal membrane offers the highest degree of structural support. These differences originate from the local chemical environment: in water, intra‐ and inter‐peptide hydrogen bonds—essential for *β*‐barrel stability—must compete with peptide–water hydrogen bonding, thereby promoting structural disassembly. Within the hydrophobic membrane core, the absence of competing H─bond donors/acceptors facilitates the maintenance of the *β*‐sheet framework. The superior stabilization afforded by the neuronal membrane is twofold. First, the liquid‐ordered phase characteristic of this multicomponent system provides a rigid scaffold that structurally reinforces the hexamer. Second, the presence of POPE facilitates favorable interactions with residues E22 and D23 in the lower headgroup region, effectively anchoring the *β*‐barrel in its transmembrane orientation. The impact of the hexameric A*β*42 *β*‐barrel on the membrane is more pronounced in the POPC bilayer than in the neuronal membrane, as evidenced by POPC flip‐flop events and drastic reductions in acyl chain order in the immediate vicinity of the peptide. This phenomenon is likely a consequence of the overall lower stability of the A*β*42–POPC system. While the phospholipids in the neuronal membrane also exhibit localized perturbations when near the barrel, the order‐providing components—specifically SM and GM1—remain relatively unaffected, maintaining the integrity of the surrounding liquid‐ordered phase.

Our results align with several experimental observations regarding the role of membrane composition in A*β*42 pathology. Previous studies have noted that while individual lipid species can either accelerate or inhibit A*β*42 aggregation [31], increased molecular complexity appears to balance these opposing effects, enhancing membrane resilience. Although our study focused on the stability of a preinserted oligomer rather than the aggregation process itself, we find that the high degree of order in the neuronal membrane confers significant resistance to the disordering effects typically induced by A*β*42. This is consistent with atomic force microscopy data showing that A*β*42 induces localized damage more effectively in ‘softer’ membranes, such as the POPC bilayers studied here [66]. However, the literature also presents a more nuanced view where certain membrane components increase vulnerability. Over 25 years ago, it was demonstrated that aggregated A*β*42 can intercalate into the hydrocarbon core of synaptic plasma membranes [67]—a scenario directly modeled in our simulations—resulting in increased membrane fluidity. Similarly, the presence of Chol has been shown to drastically increase the *β*‐sheet content of A*β*42 oligomers, significantly enhancing their toxicity compared to those formed in lipid‐free environments [68]. Our findings support this structural trend, as Chol was found to stabilize the intra‐ and inter‐peptide interactions essential for *β*‐sheet maintenance by increasing membrane stiffness. This qualitatively aligns with the experimental consensus; for instance, atomic force microscopy‐based infrared spectroscopy evaluations by Zhaliazka et al. demonstrated marked increases in aggregate *β*‐sheet content (up to 85%) within complex, Chol‐enriched lipid environments [68]. While our observed absolute values are lower, this quantitative divergence is anticipated. Our atomistic MD trajectories resolve the initial, highly dynamic conformational ensembles of early‐stage, lower‐order assemblies, whereas macroscopic spectroscopic evaluations probe highly populated, late‐stage aggregate mixtures where strong inter‐peptide coupling heavily dominates the spectral signal.

The neurotoxicity of A*β*42 is often linked to the membrane‐bound oligomer concentration, which facilitates an influx of ions (such as Ca^2+^) that triggers cellular damage [17]. A*β*42 is frequently linked to the accumulation of membrane‐bound oligomers, which facilitates an unregulated influx of extracellular ions that triggers cellular damage [17]. For instance, atomistic simulations by Yu et al. demonstrated that A*β*‐derived pentamers interact preferentially with anionic lipid matrices, where Ca^2+^ ions form stable ionic bridges between acidic peptide residues and lipid headgroups, leading to localized membrane disruption [69]. Parallel to these surface‐mediated effects, our work highlights a distinct mechanism via transmembrane channel activity. Because the embedded A*β*42 barrel exhibits superior structural stability within the neuronal membrane compared to pure POPC, we observed a continuous and consistent influx of Na^+^ ions through the pore lumen. While these channeling results suggest a direct pathway for ionic dyshomeostasis, further studies specifically tracking divalent Ca^2+^ flux within our setup are required to draw definitive conclusions regarding its relative contribution to membrane‐mediated cytotoxicity.

In summary, our simulations demonstrate that the hexameric A*β*42 *β*‐barrel is most effectively stabilized within the neuronal membrane environment, whereas it remains unstable in aqueous solution. In water, A*β*42 oligomers likely sample alternative quaternary architectures, even though the constituent *β*‐hairpin motif, derived here from NMR data [1], remains a hallmark of various A*β* oligomer species [12]. While the structural preference for pore‐forming *β*‐barrels within lipid bilayers has been previously suggested [70], the precise pathway of membrane insertion and its interplay with the aggregation process remain to be elucidated. Notably, our results reveal that A*β*42 dynamics in a complex neuronal environment differ significantly from those in a simple POPC bilayer. Therefore, we recommend that future in vitro and in silico studies focus on complex neuron‐mimicking membranes to better capture the in vivo behavior of these aggregates. Although this study focused on pre‐embedded A*β*42, these findings provide critical insights into how membrane composition dictates the stability and toxicity of amyloid aggregates. By characterizing the molecular interactions that govern these stable pore‐like structures, this work contributes to a deeper understanding of the membrane‐mediated mechanisms underlying AD.

## Conflicts of Interest

The authors declare no conflicts of interest.

## Supporting information

Supplementary Material

## Data Availability

The data that support the findings of this study are available from the corresponding author upon reasonable request.
