# Supplementary material for: Membrane Complexity and Phase Behavior Dictate the Stability of Membrane‐Inserted Aβ 42 Hexamers
Source: Chemphyschem. 2026 Aug 21;27(16):e70538. doi: 10.1002/cphc.70538 (PMC13498729; doi:10.1002/cphc.70538)
Supplement: Supplementary file 1 [file CPHC-27-e70538-s001.pdf]

Supportive Information: Membrane Complexity and Phase Behavior Dictate the Stability of Membrane-Inserted Aβ_{42} Hexamers

Bastian F. Bundschuh,[†,‡] Franziska Kley,[‡] Yu Wang,[†,‡] and Birgit Strodel[*,†,‡]

†*Institute of Biological Information Processing: Structural Biochemistry, Forschungszentrum Jülich, 52428 Jülich, Germany*

‡*Heinrich Heine University Düsseldorf, Faculty of Mathematics and Natural Sciences, 40225 Düsseldorf, Germany*

E-mail: b.strodel@fz-juelich.de

1 Supportive Information

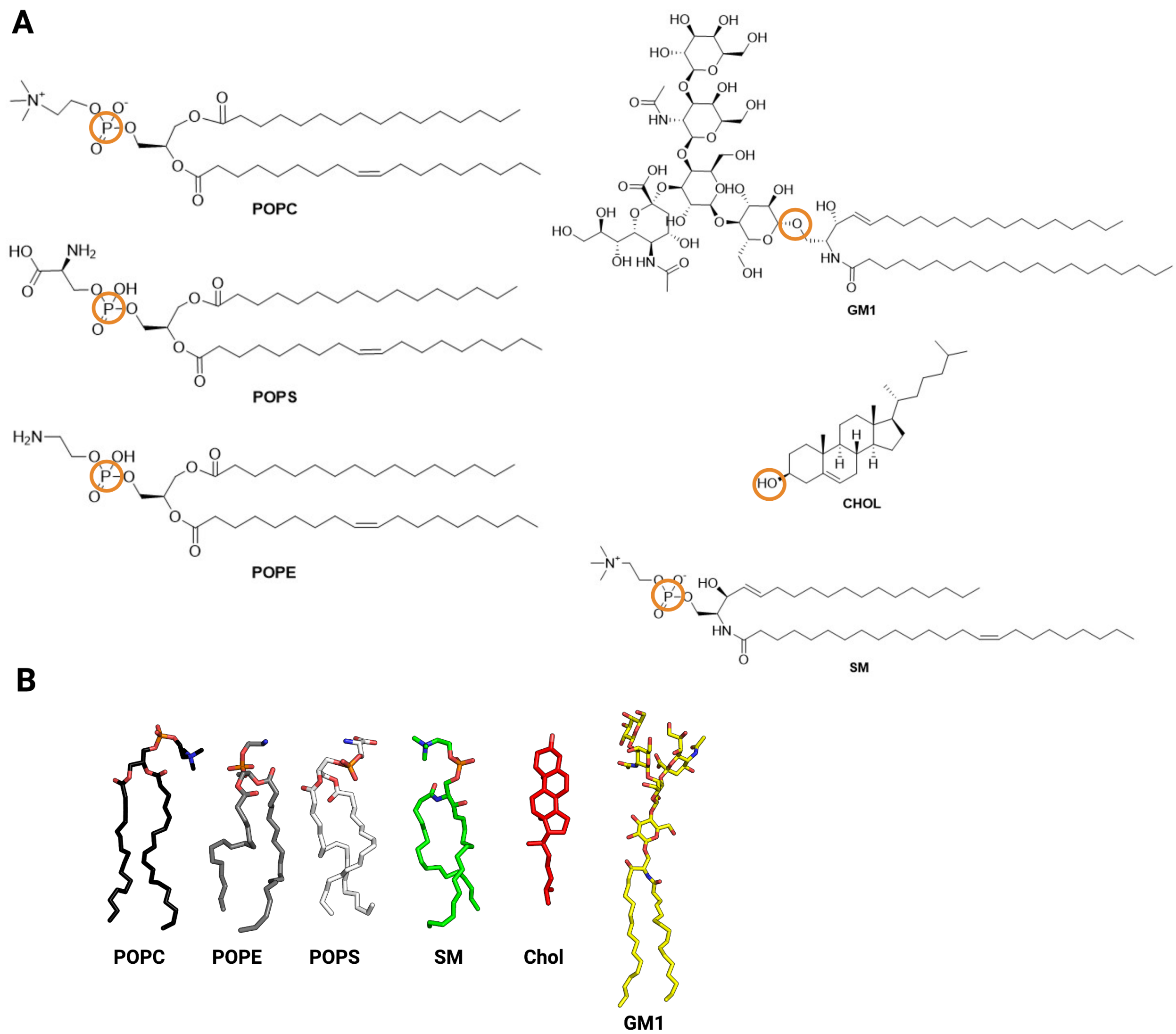

Figure S1: **Fig. S1: Chemical structures of the lipid species modeled in this work.** **A** 2D representations of the lipid species. Orange spheres highlight the phosphorus (P) atoms (or oxygen (O) atoms for Chol and GM1) used to calculate headgroup-to-headgroup distances. **B** 3D stick representations of the lipids. Carbon atoms are color-coded to distinguish between species, consistent with the color scheme used throughout the manuscript.

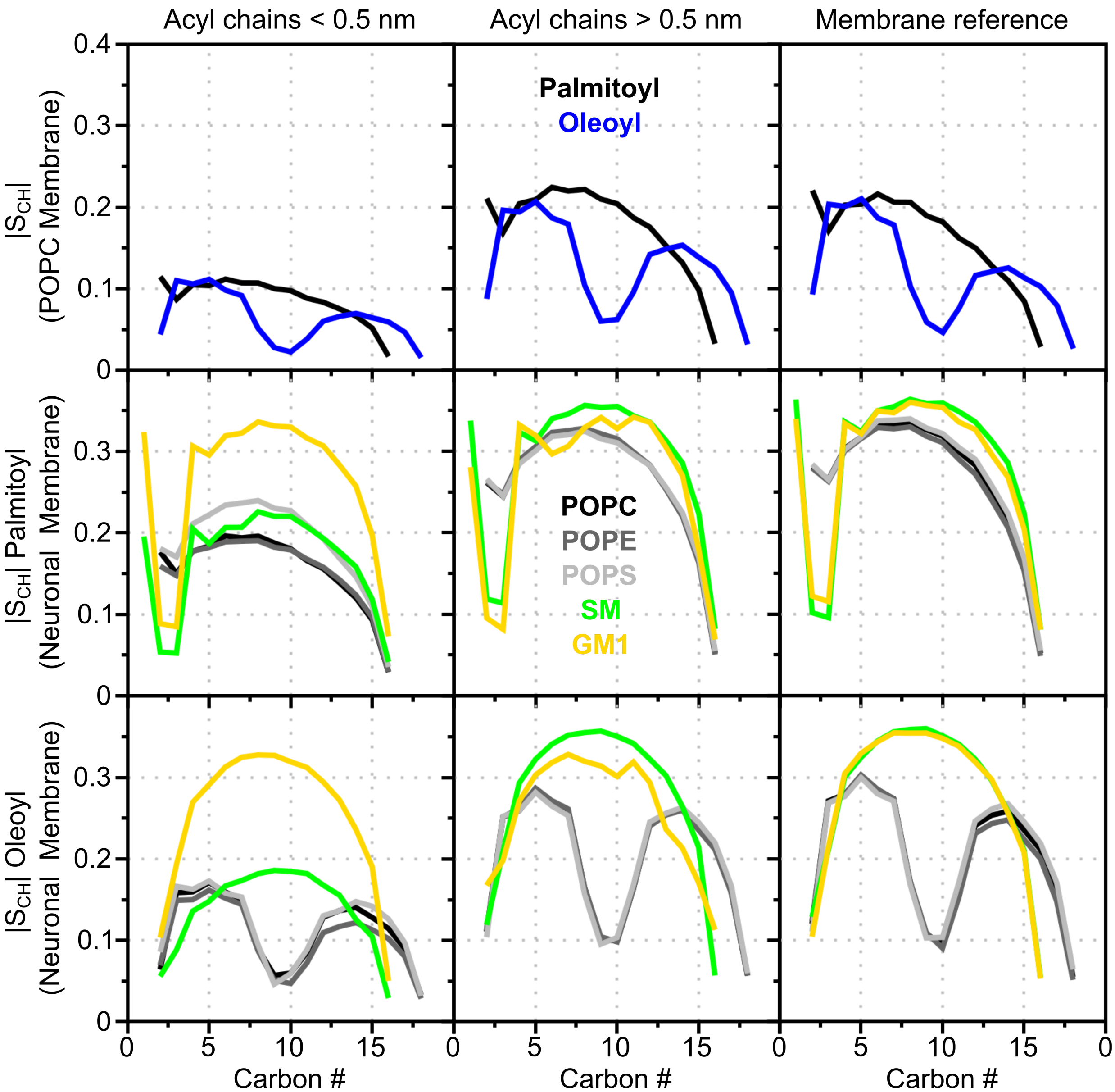

Figure S2: **Order parameters of lipid acyl chains.** Average deuterium order parameters (S_{CD}) calculated for the pure POPC and neuronal membranes. Data are shown for lipids directly interacting with the Aβ42 β-barrel ($\leq$ 0.5 nm, left), bulk lipids further from the barrel (> 0.5 nm, middle), and the unperturbed peptide-free references (right).

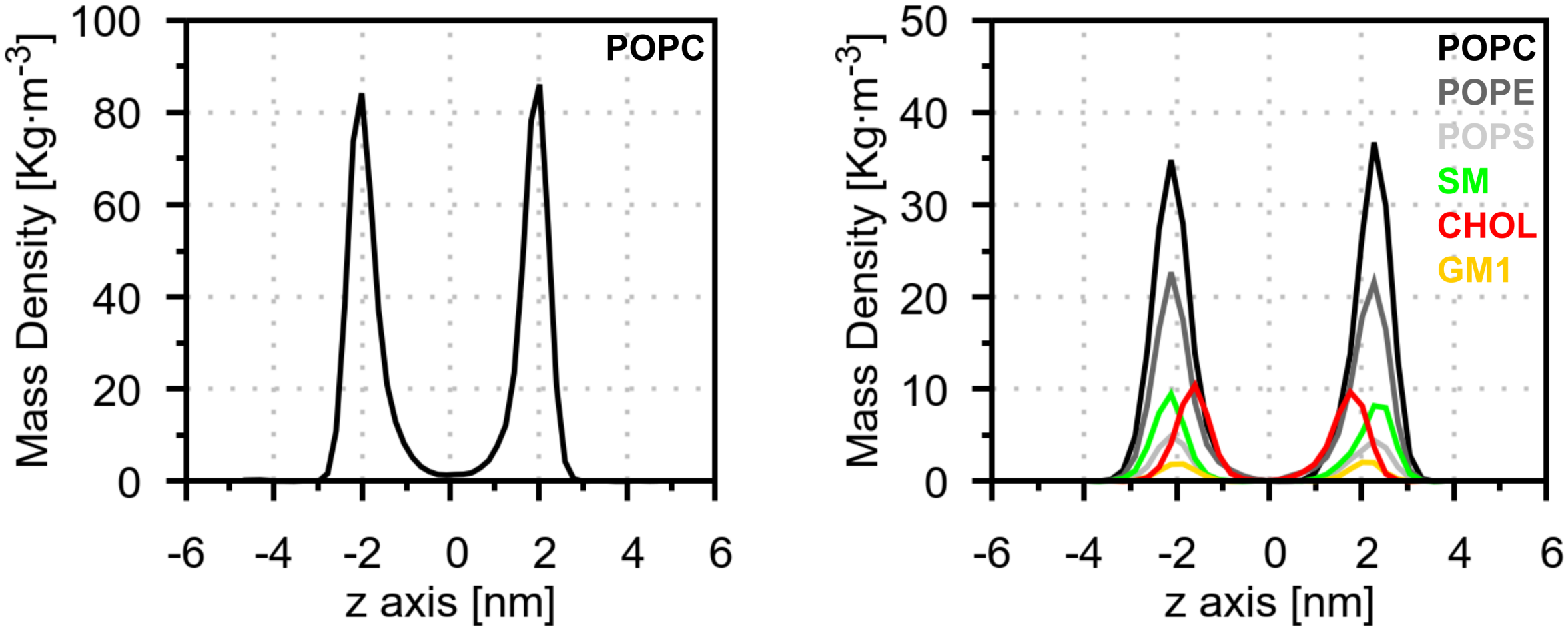

Figure S3: **Mass density profiles of membrane components.** Average mass density profiles along the bilayer normal (z-axis) for the pure POPC (left) and neuronal (right) membranes. Profiles were calculated for the lipid phosphorus (P) atoms and the oxygen (O) atoms of cholesterol and GM1, as detailed in Fig. S1. Color-coding corresponds to the legend in the upper-right corner.

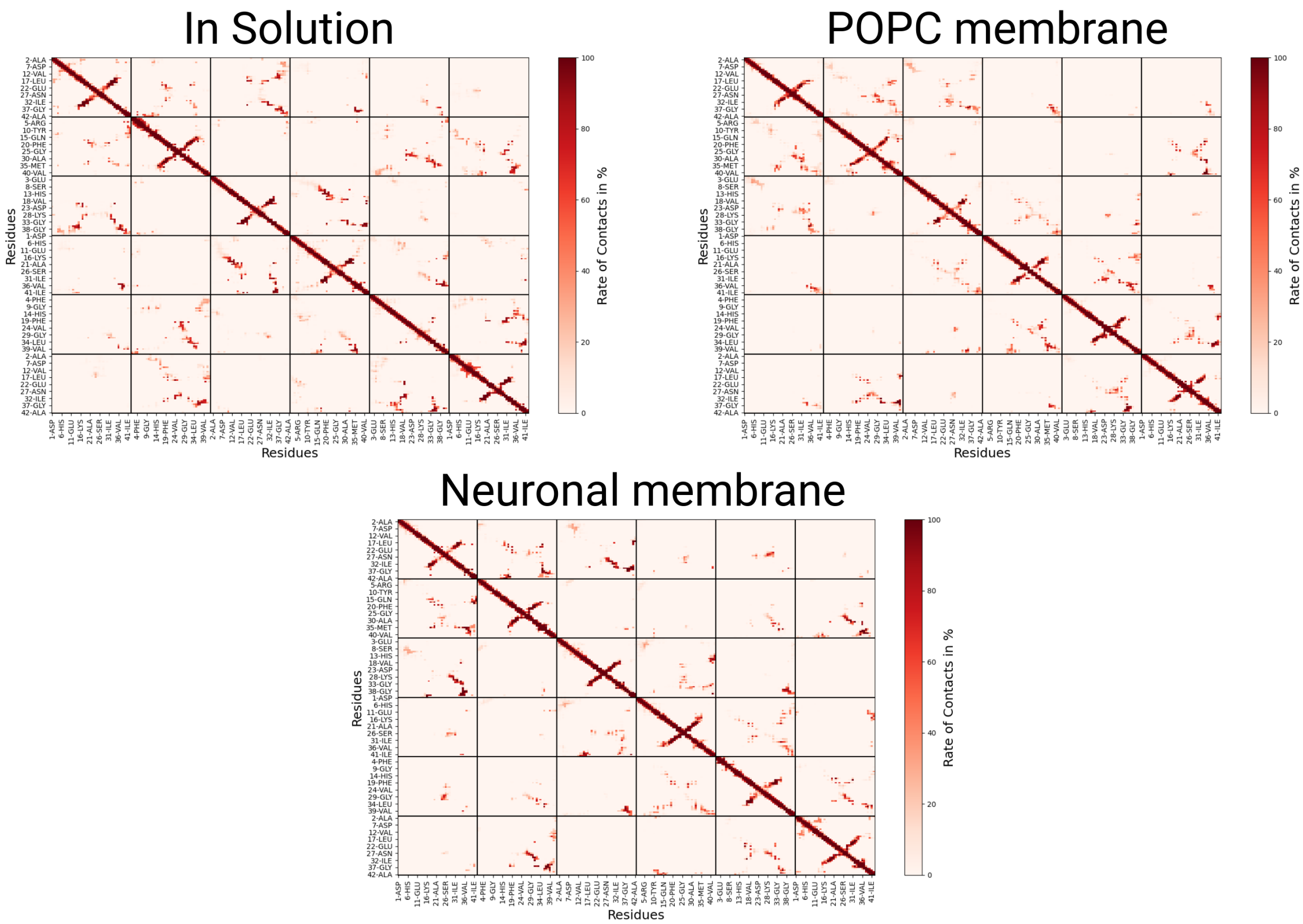

Figure S4: **Intra- and inter-peptide contact maps of the Aβ42 β-barrel in different environments.** Time-averaged residue–residue contacts are presented as heatmaps for the β-barrel simulated in solution, in a POPC membrane, and in a neuronal membrane. Intra-peptide contacts for each Aβ42 chain are shown in the diagonal blocks, while inter-peptide interactions are displayed in the off-diagonal blocks. These maps are symmetric, as the contact frequency between chains i and j is equivalent to that between chains j and i.

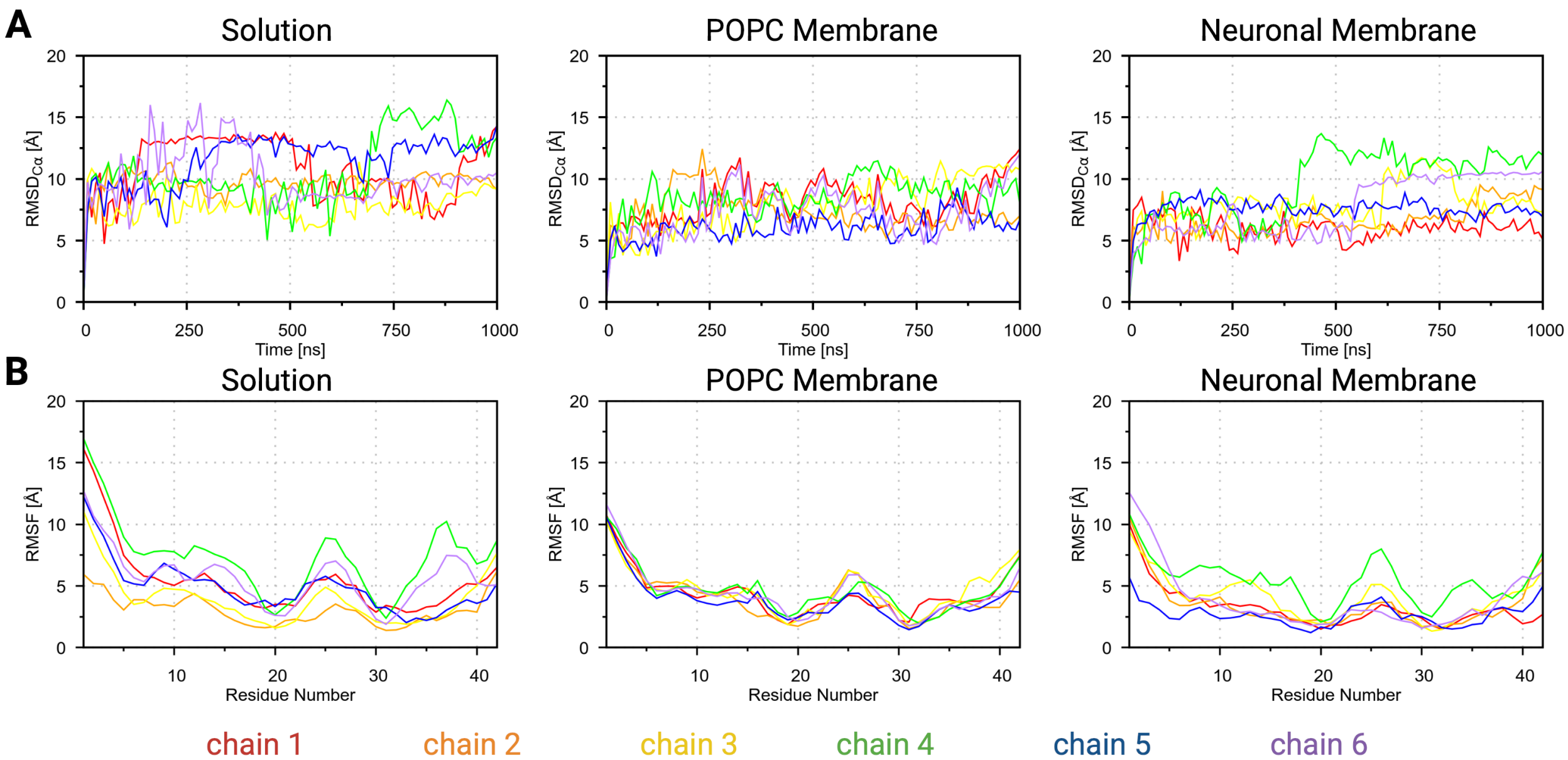

Figure S5: **Individual Aβ42 chain RMSD and RMSF profiles across all simulated environments.** **A** C_α-RMSD and **B** C_α-RMSF profiles for the six individual Aβ42 chains of the β-barrel. Results are shown for the simulations in aqueous solution, a POPC membrane, and a neuronal membrane.

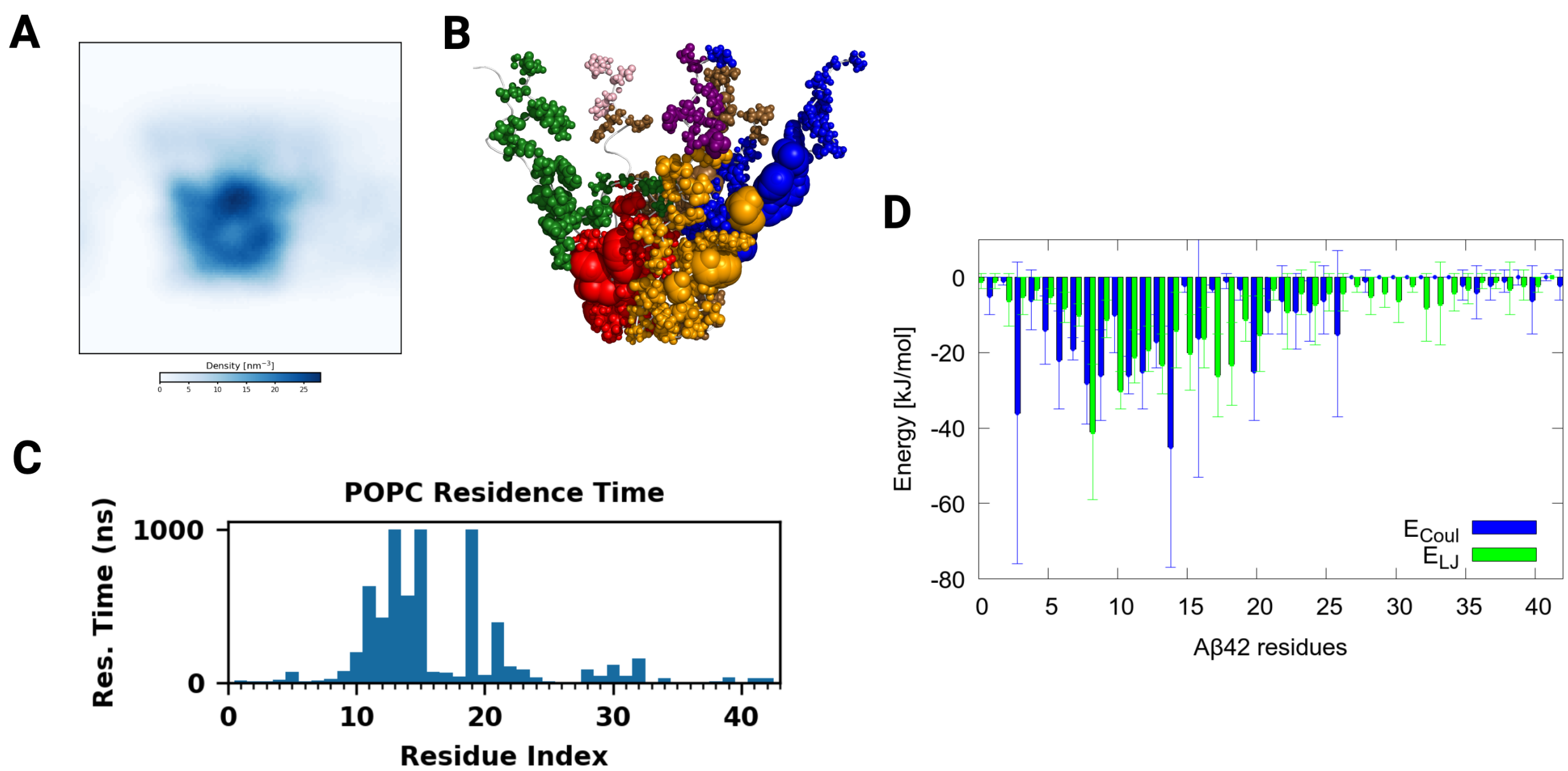

Figure S6: **Aβ42–POPC interactions within the POPC membrane system. A** Volumetric density map of the Aβ_{42} hexamer (side view). **B** The six most frequented POPC binding sites on the β-barrel, distinguished by color. Sphere sizes are proportional to the occupancy (population) within each site. **C** Residence times of POPC molecules at individual Aβ42 residues for the most highly populated binding site. **D** Time-averaged POPC–Aβ42 interaction energies, with fluctuations indicated by minimum and maximum values. Energies are decomposed into Coulombic (blue) and Lennard-Jones (green) contributions.

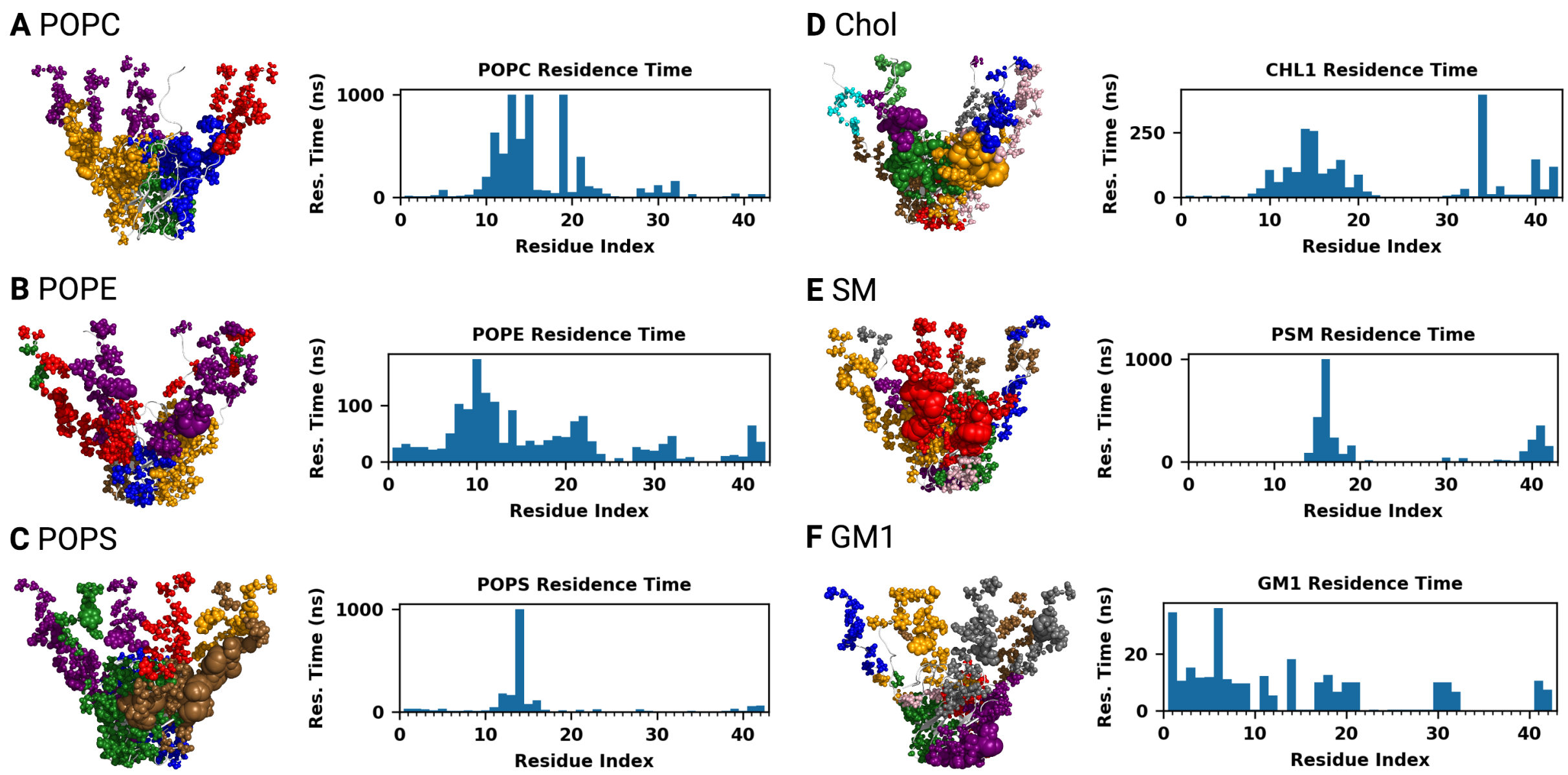

Figure S7: **Lipid binding sites at Aβ42 and residence times for the neuronal membrane simulation.** The six most frequented binding sites for **A** POPC, **B** POPE, **C** POPS, **D** Chol, **E** SM, and **F** GM1 of the neuronal membrane are shown on the β-barrel, distinguished by color. Sphere sizes are proportional to the occupancy within each site. Accompanying plots on the right of each panel display the residence times at individual Aβ42 residues for the most highly populated binding site of the respective lipid species.
